# Enabling doctor-centric medical AI with LLMs through workflow-aligned tasks and benchmarks

**DOI:** 10.1038/s44401-025-00038-z

**Published:** 2025-11-25

**Authors:** Wenya Xie, Qingying Xiao, Yu Zheng, Xidong Wang, Junying Chen, Ke Ji, Anningzhe Gao, Prayag Tiwari, Xiang Wan, Feng Jiang, Benyou Wang

**Affiliations:** 1 School of Data Science, The Chinese University of Hong Kong, Shenzhen, Shenzhen, Guangdong China; 2 Shenzhen Research Institute of Big Data, Shenzhen, Guangdong China; 3 National Health Data Institute, Shenzhen, Shenzhen, Guangdong China; 4 Halmstad University, Halmstad, Halland Sweden; 5 Shenzhen University of Advanced Technology, Shenzhen, Guangdong China

**Keywords:** Data integration, Data mining

## Abstract

The rise of large language models (LLMs) has transformed healthcare by offering clinical guidance, yet their direct deployment to patients poses safety risks due to limited domain expertise. To mitigate this, we propose repositioning LLMs as clinical assistants that collaborate with experienced physicians rather than interacting with patients directly. We conducted a two-stage inspiration–feedback survey to identify real-world needs in clinical workflows. Guided by this, we constructed DoctorFLAN, a large-scale Chinese medical dataset comprising 92,000 Q&A instances across 22 clinical tasks and 27 specialities. To evaluate model performance in doctor-facing applications, we introduced DoctorFLAN-test (550 single-turn Q&A items) and DotaBench (74 multi-turn conversations). Experimental results with over ten popular LLMs demonstrate that DoctorFLAN notably improves the performance of open-source LLMs in medical contexts, facilitating their alignment with physician workflows and complementing existing patient-oriented models. This work contributes a valuable resource and framework for advancing doctor-centered medical LLM development.

## Introduction

Large Language Models (LLMs) have demonstrated significant potential in various applications within healthcare, such as autonomous online consultations, which can reduce costs and improve accessibility to medical services^1–8^. However, using LLMs as a direct consulting tool for patients can bring serious health risks because patients lacking medical expertise are easily misled by the inaccurate medical advice generated by the model^9–11^.

In contrast, developing LLMs as medical assistants for healthcare professionals presents a safer and more practical direction. Doctors routinely deal with complex information processing tasks, such as summarizing patient records, providing clinical decision support, and educating patients. Using LLMs for these tasks could significantly alleviate the workload of doctors, allowing them to perform their duties more efficiently^12,13^. Furthermore, LLMs have shown promising results in multi-task settings^14,15^, suggesting that LLMs have substantial potential when applied to a multi-functional medical assistant role. Despite these promising developments, there remains a significant gap between the current capabilities of LLMs and the complex requirements of real-world medical practice. Most existing medical LLMs^3,6,16–18^ have been trained on patient-centric datasets, which focus primarily on tasks like pre-diagnosis and medical consultation. These datasets are limited in scope and do not encompass the diverse and multifaceted nature of clinical tasks encountered in actual medical environments. Moreover, previous research on LLMs as medical assistants has often focused on a narrow set of tasks^12,13^, and these models frequently fail to provide comprehensive responses to complex, real-world medical inquiries^19,20^. Another critical limitation lies in the current benchmark tests, which often do not adequately assess the performance of LLMs as medical assistants. Most widely used benchmarks rely on multiple-choice question formats^1,10,19–22^, which fail to align with the real-world requirements where detailed and comprehensive responses are needed. Alternatively, these benchmarks typically assess only a small subset of tasks^1^, failing to cover the full range of workflows that doctors encounter in practice.

**Fig. 1:**
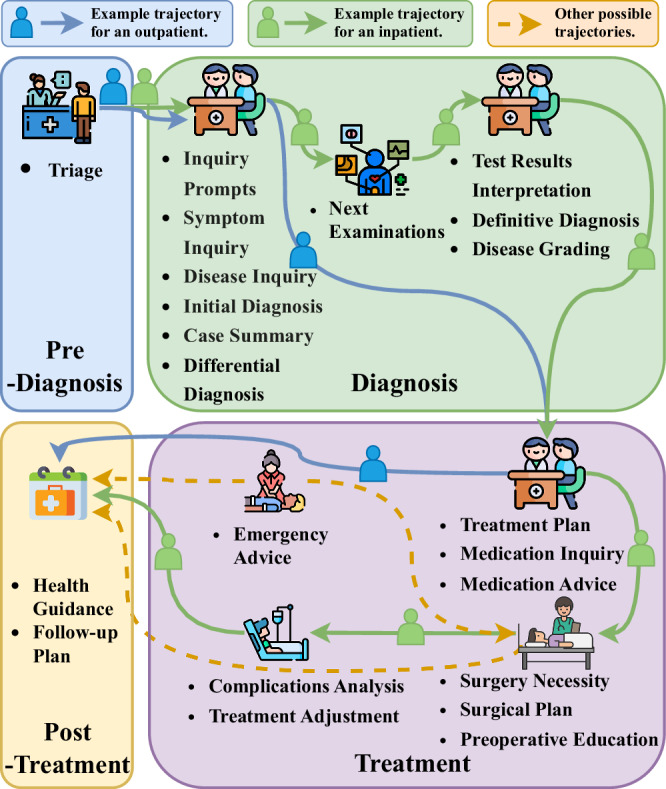
Task categories finalized for llms in medical assistance, organized by four phases: pre-diagnosis, diagnosis, treatment, and post-treatment.

To address the above issues, we aim to develop LLMs as better doctor assistants by building comprehensive and practical datasets and evaluations. Firstly, to gain a thorough understanding of doctors’ needs for medical assistants, we collaborate with dozens of professional doctors to explore 22 tasks across four phases in real-world scenarios. These tasks are finalized through a two-stage survey using a heuristic-feedback method, as shown in Fig. 1. Based on these insights, we develop DoctorFLAN, a comprehensive Chinese medical dataset containing approximately 92K samples that capture the full spectrum of the doctor’s daily work, including both inpatient and outpatient scenarios. It leverages GPT-4-polishing with reference enhancement, followed by manual verification from professional doctors, to ensure samples provide reliable and comprehensive expert responses for training our model (DotaGPT).

To develop effective doctor assistants, we construct a novel benchmark for medical LLMs that includes both single-turn evaluation (DoctorFLAN-*test*) and multi-turn evaluation (DotaBench) by simulating the dialog with doctors in receiving patients scenarios. The existing popular LLMs and our model, DotaGPT, are evaluated both automatically and manually on the benchmark. The results indicate that existing models, while acting as virtual doctors assisting patients, struggle with the diverse and complex tasks required for real-world roles that assist doctors. In contrast, our DoatGPT serving as a doctor assistant exhibits robust performance across tasks in both DoctorFLAN-*test* and DotaBench.

Our contributions are threefold:

1. We explore the underexplored scenario of developing medical models as doctor assistants, providing essential data, models, and benchmarks that complement existing research in this domain.
2. We construct a 92K-sample dataset for doctor assistants by collaborating with dozens of medical professionals, using a heuristic-feedback method to identify 22 key tasks and employing reference-enhanced polishing and manual verification.
3. We introduce an expert-involved benchmark to assess LLMs in doctor-oriented scenarios, covering both single-turn and multi-turn interactions, and thoroughly analyze the consistency between manual and automatic evaluations, comparing them with widely accepted benchmarks.

## Results

### Automatic evaluation results

Table 1 outlines the automatic evaluation results of the existing medical models on DoctorFLAN-*test*.

**Table 1:** Automatic evaluation results on DoctorFLAN-*test* and DotaBench

Model	Size	DoctorFLAN-*test*					DotaBench
		Pre-diagnosis	Diagnosis	Treatment	Post-treatment	Average	Average
Open-source general LLMs							
Qwen-1.8B-Chat	1.8B	5.28	4.56	3.96	5.44	4.48	4.68
Baichuan-13B-Chat	13B	6.20	6.51	6.31	7.55	6.57	7.59
Baichuan2-7B-Chat	7B	6.32	6.36	6.34	7.70	6.59	7.41
Baichuan2-13B-Chat	13B	6.76	6.85	6.94	7.81	7.04	7.47
Yi-6B-Chat	6B	7.00	6.83	6.83	7.66	6.98	8.25
Yi-34B-Chat	34B	7.36	7.38	7.95	8.78	7.80	8.65
Open-source medical LLMs							
BianQue-2	6B	5.56	3.27	3.65	4.78	3.72	4.12
DISC-MedLLM	13B	5.56	4.23	3.54	5.14	4.24	4.97
HuatuoGPT	7B	5.32	4.24	3.72	4.92	4.29	5.88
HuatuoGPT-II	7B	7.60	7.02	6.69	7.42	7.03	7.90
DotaGPT_Yi-6B_	6B	8.32	7.62_*↑* 11.6%_	7.68_*↑* 12.4%_	8.44	7.81_*↑* 11.9%_	8.36 _*↑* 1.3%_
DotaGPT_Baichuan2-7B_	7B	8.48	8.01_*↑* 25.9%_	8.23_*↑* 29.8%_	8.80	8.25_*↑* 25.2%_	_*↑* 12.8%_
Proprietary LLMs							
GPT-3.5	N/A	6.40	6.85	6.26	6.74	6.64	7.83
Claude-3	N/A	7.80	8.38	8.28	8.76	8.38	9.21
GPT-4	N/A	8.00	8.41	8.28	9.04	8.42	9.41

The subscript of DotaGPT (e.g., DotaGPT_Yi-6B_) indicates the backbone on which the model was initially trained. The red arrows (*↑*) with percentages indicate the improvement of DotaGPT over the corresponding chat models with the same backbone.

#### Take-away 1

Existing models perform poorly in the diagnosis and treatment phases.

The results reveal a notable performance decline for all models during the diagnosis and treatment phases compared to the pre-diagnosis and post-treatment phases. This drop may be attributed to the high medical knowledge requirements of tasks like *Disease Grading* and *Surgical Plan*, for which models are often undertrained due to a lack of knowledge-intensive datasets. However, DotaGPT models show a significant improvement in these phases. Specifically, DotaGPT_Baichuan2-7B_ and DotaGPT _Yi-6B_ exhibit performance increases of 11.6% and 12.4% in the diagnosis phase, and 25.9% and 29.8% in the treatment phase, respectively. These enhancements demonstrate the value of our tailored dataset in improving performance on complex medical tasks.

#### Take-away 2

Larger models perform better.

When comparing Yi-6B-Chat (average score: 6.98) with Yi-34B-Chat (average score: 7.80), and Baichuan2-7B-Chat (average score: 6.59) with Baichuan2-13B-Chat (average score: 7.04), we observe that larger models consistently outperform their smaller counterparts across all four phases. The models with more parameters achieve higher average scores, likely due to their enhanced reasoning abilities, which better equip them to handle the considerable complexity of the tasks in our evaluation.

#### Take-away 3

Limitations of virtual doctor models in workflow assistance tasks.

Virtual doctor models originally designed to provide medical advice to patients, such as BianQue-2 and HuatuoGPT, perform relatively poorly in tasks related to doctor workflow assistance, with scores of 4.12 and 5.88, respectively. These models are primarily trained on large medical dialogue datasets, where the focus is on mimicking the question-and-answer style of doctors, with the goal of functioning as a virtual doctor. However, medical dialogues like Huatuo26M^23^ are mostly based on online consultations, which may not capture the full range of tasks involved in a doctor’s workflow. As a result, these models struggle with more specific, nuanced tasks that occur in everyday medical practice.

#### Take-away 4

Medical dataset fine-tuning does not always enhance performance on DoctorFLAN.

A comparison between the DISC-MedLLM (4.24) and its chat counterpart, Baichuan-13B-Chat (6.57), reveals that the medical domain-specific fine-tuning of DISC-MedLLM does not lead to better performance on the DoctorFLAN tasks. In fact, the fine-tuned DISC-MedLLM underperforms compared to the general-purpose Baichuan-13B-Chat. This outcome underscores the potential risks of excessive specialization, suggesting that a balance between domain-specific fine-tuning and general adaptability is crucial for ensuring broader model applicability.

#### Take-away 5

DoctorFLAN fine-tuning improves performance on doctor-assistance tasks.

In contrast, our DotaGPT variants, fine-tuned on the DoctorFLAN dataset, demonstrate significant performance improvements over their respective chat model counterparts. Specifically, the variant fine-tuned on Baichuan2-7B shows a substantial improvement of 25.2%. Similarly, the DotaGPT variant fine-tuned on Yi-6B outperforms the Yi-6B-Chat by 11.9%. The improvement on both backbones highlights the effectiveness of DoctorFLAN and brings our models’ performance close to those of leading proprietary models such as Claude-3 and GPT-4.

We further evaluate DotaGPT’s performance on DotaBench to assess its ability in practical multi-turn settings, which reflects its real-world applicability. This out-of-domain evaluation is detailed in Table 1. Notably, our DotaGPT variants significantly outperform models of comparable size on DotaBench, even surpassing the larger Yi-34B-Chat model. This strong performance underscores DotaGPT’s robust ability to generalize from DoctorFLAN to out-of-domain contexts Table 2.

**Table 2:** Human evaluation results on DoctorFLAN-*test*

Models	Average Score
BianQue-2	4.58
HuatuoGPT	4.97
DISC-MedLLM	5.36
Baichuan2-7B-Chat	6.69
GPT-4	8.06
DotaGPT_Baichuan2-7B_	7.83

For detailed task-by-task results.

### Human evaluation results

Aside from the automatic evaluation, we conducted manual evaluation on a subset of models due to resource constraints, as shown in Tables 2 and 3. The results show that on DoctorFLAN-*test*, DotaGPT_Baichuan2-7B_ (7.83) outperforms patient-assistance models like BianQue-2 (4.58), HuatuoGPT (4.97), and DISC-MedLLM (5.36), as well as the general counterpart Baichuan2-7B-Chat (6.69), consistent with the automatic evaluation results. Further, human evaluation results on DotaBench, shown in Table 3, confirm DotaGPT_Baichuan2-7B_’s strong performance, with an average score of 8.54, surpassing Baichuan2-7B-Chat (8.25) by 3.5%.

**Table 3:** Human evaluation results on the DotaBench

Model	Average score
Baichuan2-7B-Chat	8.25
DotaGPT_Baichuan2-7B_	8.54_*↑* 3.5%_

**Fig. 2:**
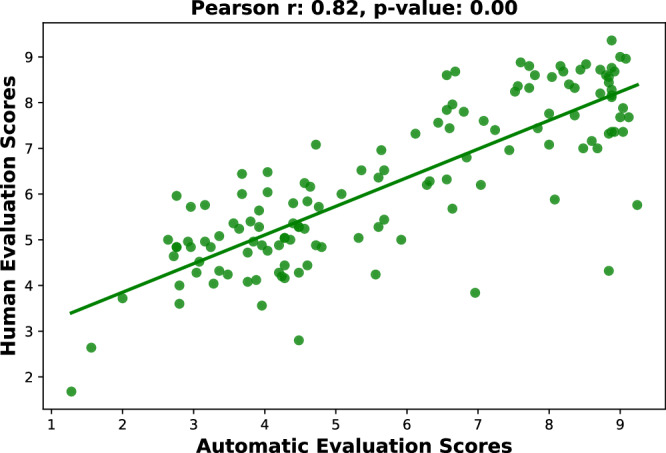
Correlations between human and automatic evaluations on DoctorFLAN-test, illustrating task-level consistency.

To verify the reliability of our evaluation methods, we also conducted a task-level correlation analysis between human and automatic evaluations on the DoctorFLAN-*test*. For each model and task, we averaged the results across 25 samples per task (this averaging is done to ensure consistency and minimize the impact of outliers or variance in individual responses). Our analysis, covering 132 data points, reveals a Pearson correlation coefficient of 0.82, indicating strong consistency between evaluation modes^24^, as shown in Fig. 2.

### Generalization of DotaGPT on other benchmarks

To further evaluate DotaGPT’s medical knowledge and generalization capability, we assess its performance on several established medical benchmarks, as shown in Table 4. DotaGPT_Baichuan2-7B_ delivers competitive results across CMMLU^25^, MMLU^26^, CMExam^27^, and CMB-Exam^10^. Notably, it outperforms Baichuan2-7B-Chat in 3 out of 4 categories. Although DotaGPT_Baichuan2-7B_ falls short of HuatuoGPT-II^5^, this performance gap may be attributed to the significantly larger training dataset used by HuatuoGPT-II.

**Table 4:** Comparative performance of medical LLMs on diverse medical benchmarks

Model	CMMLU_*Med*._	CMExam	MMLU_*Med*._	CMB-exam
Open-source medical LLMs				
DISC-MedLLM	-	36.62	-	32.47
HuataoGPT-II	59.08	65.81	51.44	59.00
Baichuan2-7B-Chat	50.74	50.48	50.29	43.33
DotaGPT_Baichuan2-7B_	54.58	59.76	48.49	52.42^*^
Proprietary LLMs				
GPT-4	-	-	-	59.46

CMB-exam scores are from ref. ^10^, except for DotaGPT_Baichuan2-7B_^*^.

### Case study

To provide a clearer demonstration of our model’s ability to generate knowledge-intensive responses in doctor-oriented tasks, we selected a case from *Differential Diagnosis* for comparison. As detailed in Table 5, we presented the responses from both HuatuoGPT and DotaGPT(Baichuan2-7B). Despite HuatuoGPT’s fluent responses, they lack substantial information, often repeating general rather than medically specific answers. Conversely, DotaGPT not only accurately diagnosed the condition but also provided detailed, professional reasoning, demonstrating its superior ability to deliver knowledge-intensive answers.

**Table 5:**
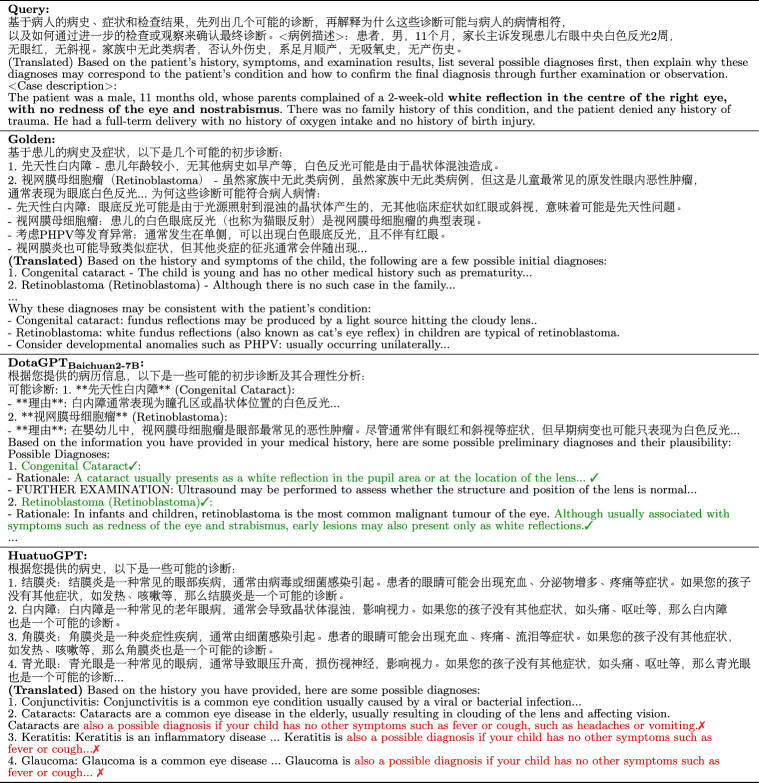
Illustrative case study from the *Initial Diagnosis* task in DoctorFLAN-*test*, showing Chinese model responses along with key English highlights for clarity

The example includes the ground truth (Golden) and outputs from DotaGPT_Baichuan2-7B_ and HuatuoGPT. We annotate key segments using green *✓* for medically correct information and red ✗ for incorrect or irrelevant reasoning. The full Chinese outputs are preserved to support fine-grained comparison across models.

## Discussion

In this paper, we focus on underexplored scenarios of developing medical models as doctor assistants. We first collaborated with dozens of doctors and conduct a two-stage survey to accurately identify real-world clinical tasks for efficient doctor assistance. We then created DoctorFLAN, using reference-enhanced refinement to overcome the training limitations of previous models. Additionally, we introduced DotaBench as a complementary evaluation to assess the effectiveness of popular medical LLMs as doctor assistants. Benchmark results indicate that while existing LLMs face challenges in this role, DotaGPT’s performance shows that our dataset can significantly enhance their capability, providing a valuable supplement to current medical LLM research.

We also acknowledge the following limitations of this stage work. The DoctorFLAN is currently only available in Chinese and may require supplementation in other languages. Consequently, it cannot be guaranteed that DotaGPT trained on DoctorFLAN will perform well in languages other than the one on which it has been tested. However, the methodology employed to create DoctorFLAN can be applied universally across different languages. Additionally, although DotaGPT has demonstrated impressive performance on the benchmarks, it is important to exercise caution when using its outputs, particularly in real-world doctor-oriented interactions.

Ensuring the privacy and security of data is paramount in the development of medical applications. The datasets used in this study, primarily derived from Medtiku^28^, an open-source repository of medical examination questions, and PromptCBLUE^29^, are both freely available for use. Additionally, we incorporated data from an internet medical encyclopedia hosted by 120 Ask^30^, also open to the public.

Given the potential issues with the credibility of content generated by DotaGPT, we are committed to strictly regulating the model’s use to prevent misuse. Our datasets, DoctorFLAN and DotaBench, have been released under terms that uphold the highest ethical standards. This commitment ensures that while advancing the capabilities of large language models in healthcare, we also safeguard sensitive medical data.

## Methods

### Necessity of LLMs for doctors

Recent advancements in large medical LLMs such as PMC-LLaMA^31^, Med-PaLM^1^, Med-PaLM2^2^, and HuatuoGPT-II^5^ have significantly contributed to enhancing the domain-specific knowledge of these models and have supported the subsequent application of medical LLMs. Leveraging these advancements, several popular medical application models^3,6,7,32–34^ are trained on extensive patient-doctor dialogues with the goal of functioning as autonomous virtual doctors, providing medical consultations directly to patients.

Despite advancements, the accuracy of these models in generating expert-level medical advice remains insufficient^11^. Directly providing their responses to patients without medical training poses significant risks, as these patients may not be able to identify errors. For instance, a patient with suspected appendicitis presenting with abdominal pain and fever may receive an incomplete recommendation from the model, potentially delaying critical intervention.

In contrast, healthcare professionals, equipped with specialized medical knowledge, are capable of identifying such errors. This highlights the potential of developing large medical language models designed to assist doctors in addition to direct patient consultation. While recent efforts have been made to develop medical LLMs as assistants to support doctors on specific scenarios, such as MedDM^12^ for differential diagnosis and treatment recommendations and Dia-LLaMA^13^ for CT report generation. However, these works typically address only isolated tasks, leaving a significant gap in the development of LLMs capable of comprehensively supporting the full spectrum of tasks within a doctor’s workflow.

### Towards better doctor assistants

Developing a medical LLM capable of assisting across the entire clinical workflow requires a dataset that comprehensively covers all relevant tasks while providing detailed and accurate responses. Furthermore, a practical benchmark is essential to evaluate whether the model can generate outputs that effectively support doctors in real-world scenarios.

#### Training data across the entire workflow

As shown in Table 6, existing datasets for online medical consultation dialogues, such as Huatuo-26M^23^, MedDialog^16^, and others^3,17,18^, primarily provide responses for pre-diagnosis scenarios. However, these datasets only cover a limited portion of medical scenarios, making them unsuitable for comprehensive, end-to-end medical workflows. Conversely, structured resources such as knowledge graphs (e.g., CMeKG^19^) and multiple-choice question answer datasets (e.g., MedMCQA^20^ and CMExam^27^) cover a broader range of clinical scenarios but are limited in their ability to generate knowledge-intensive, context-rich responses. Thus, there is an urgent need for a comprehensive dataset that not only encompasses the entire spectrum of a doctor’s workflow but also provides detailed and context-rich answers. Such a dataset is crucial for effectively training and deploying LLMs in clinical settings.

**Table 6:** Comparison of existing medical training datasets

Dataset	Applied scenarios	Entire workflow	Knowledge-intensive responses
Huatuo-26M	OMCD	✗	*✓*
MedDialog	OMCD	✗	*✓*
HealthCareMagic100k	OMCD	✗	*✓*
ChatDoctor10k	OMCD	✗	*✓*
webMedQA	OMCD	✗	*✓*
KUAKE-QIC	OMCD	✗	*✓*
CMeKG	KG	*✓*	✗
CMExam	MCQA	*✓*	✗
MedMCQA	MCQA	*✓*	✗
DoctorFLAN &DotaBench	DAQA	*✓*	*✓*

*OMCD* Online Medical Consultant Dialogue, *KG* Knowledge Graph, *MCQA* multiple-choice question answer, *DAQA* doctor-oriented Question Answer.

#### Doctor-assistance benchmark for clinical workflows

Furthermore, existing benchmarks are insufficient for effectively evaluating models as medical assistants due to their lack of alignment with practical, real-world scenarios. Common benchmarks, such as PubMedQA^21^, MedQA^22^, MultiMedQA^1^, MedMCQA^20^, CMExam^27^, and CMB^10^, primarily focus on assessing knowledge accuracy through multiple-choice questions. However, real-world medical tasks are rarely limited to answering multiple-choice questions. Instead, they often require more nuanced decision-making accompanied by detailed analysis and explanations. Similarly, benchmarks like PromptCBLUE^29^, which evaluate isolated skills such as Named Entity Recognition in medical NLP tasks, fail to capture the integrated and contextually rich requirements of doctor-assistant applications. While open-ended benchmarks like HealthSearchQA^1^ offer broader evaluations, they still fall short of covering the full spectrum of tasks encountered in a doctor’s workflow. Thus, there is a clear need for more realistic and comprehensive benchmarks that accurately simulate diverse medical practice scenarios. These benchmarks should be designed to evaluate the ability of LLMs to function as effective doctor assistants, providing contextually aware, detailed, and practical responses that align with real-world requirements.

### Task and dataset development for clinical workflows

Prior clinical NLP systems, such as cTAKES^35^ have primarily focused on retrospective information extraction, aiming to standardize clinical notes through rule-based processing for tasks like concept normalization and coding. We shifted the focus from retrospective extraction to prospective generation, designing workflow-aligned, open-ended tasks that reflect real-world clinical needs. To support workflow-aligned generation, we first defined a set of 22 representative tasks that spanned the entire clinical workflow. These tasks were derived through expert interviews and validated via a large-scale survey with licensed physicians to ensure their practical relevance and generalizability. Building on this task framework, we constructed two complementary datasets: DoctorFLAN, which covers single-turn Q&A aligned with each task, and DotaBench, which extends the task design into multi-turn dialogue settings.

To ensure that the tasks identified aligned closely with the practical needs of medical professionals, we organized a symposium with 16 medical experts to discuss key tasks in the medical workflow. To avoid omissions, the experts categorized the workflow into four phases: Pre-diagnosis, Diagnosis, Treatment, and Post-treatment. In each phase, the experts identified and outlined the specific tasks that doctors typically perform in daily practice.

Pre-diagnosis tasks are actions that doctors perform before the diagnostic process. The tasks identified in this phase include *Triage*, as outlined in Table 7. Compared to the diagnostic and treatment tasks, the pre-diagnosis tasks generally involve fewer complex medical decisions. However, the introduction of LLMs has the potential to enhance workflow efficiency by automating the generation of simple decision-making outcomes.

**Table 7:** Tasks identified in the four phases

Phase	Specific tasks	Detailed description
Pre-diagnosis	Triage	Recommend suitable departments based on patient symptoms
Diagnosis	Inquiry Prompts	Suggest follow-up questions based on patient history
	Symptom Inquiry	Provide key information about specific symptoms
	Disease Inquiry	Provide key information about specific diseases
	Initial Diagnosis	Identify possible conditions based on initial assessments
	Case Summary	Compile key points from doctor-patient dialogue into a patient case
	Differential Diagnosis	Differentiate between conditions with similar symptoms
	Next Examinations	Recommend necessary tests for further clarity
	Test Results Interpretation	Explain the implications of test results
	Definitive Diagnosis	Confirm the most likely diagnosis
	Disease Grading	Categorize disease severity using standard criteria
Treatment	Emergency Advice	Provide guidance for urgent medical situations
	Treatment Plan	Propose potential treatment approaches
	Medication Inquiry	Offer detailed information about medications
	Medication Advice	Provide specific medication recommendations
	Complications Analysis	Highlight potential risks or complications
	Treatment Adjustment	Recommend updates based on patient response
	Surgery Necessity	Assess the need for surgical intervention
	Surgical Plan	Outline key considerations for surgery
	Preoperative Education	Explain surgery and postoperative care to patients
Post-treatment	Health Guidance	Advise on recovery and recurrence prevention
	Follow-up Plan	Develop a plan for regular check-ups and ongoing care

Diagnosis tasks encompass all activities performed by doctors during the diagnostic process that contribute to formulating the final diagnosis. The tasks are summarized in Table 7. Given the complexity of medical decision-making in this phase, LLMs have significant potential to assist doctors in improving decision quality. For example, in the questioning prompts task, LLMs can generate questions based on the patient’s condition, encouraging doctors to conduct more comprehensive and thorough inquiries. In clinical practice, less experienced doctors may overlook critical diagnostic considerations, failing to take a complete medical history. LLMs can alleviate this by providing additional prompts that guide thorough questioning. For instance, when evaluating a patient with abdominal pain, some doctors may focus solely on the location and intensity of pain, while an LLM might prompt the doctor to inquire about changes in bowel habits, potentially revealing diagnostic clues such as irritable bowel syndrome or inflammatory bowel disease. Additionally, some tasks, such as *Case Summary*, can enable LLMs to automatically generate medical case summaries, thereby saving time and effort.

Treatment tasks refer to all actions performed by doctors after diagnosis and before patient discharge. These tasks include outpatient tasks such as *Medication Advice* and inpatient tasks such as *Surgical Plan*, with a complete task definition provided in Table 7. LLMs have the potential to assist doctors in these tasks by providing advice, thereby improving decision accuracy and consistency.

Post-treatment tasks are those that occur after a patient has completed their primary treatment and is transitioning to long-term recovery or ongoing management. The tasks in this phase primarily involve *Health Guidance* and *Follow-up Plan*, as detailed in Table 7. While long-term management tasks involve fewer complex decisions, they still require considerable time and effort from doctors. LLMs can help by quickly generating suggestions, improving workflow efficiency in this phase.

### Validating the task coverage through expert collaboration

**Fig. 3:**
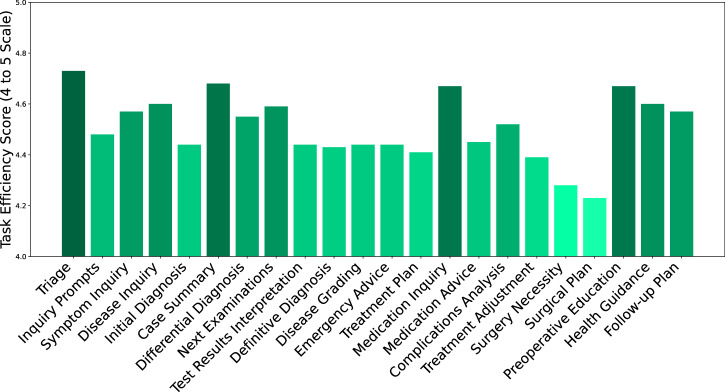
Comparative assessment of task efficiency scores for each task according to our survey.

To further validate the universality of the tasks defined in the focus group discussions and gain deeper insights into doctors’ needs for medical LLM assistance, we conducted a survey with doctors from 13 tertiary hospitals. To ensure respondent qualifications, we distributed the survey exclusively within verified professional groups composed of licensed, practicing physicians with relevant clinical experience. The survey does not collect any personally identifiable information in order to respect respondent privacy and encourage candid feedback. We initially listed all 22 predefined tasks and ask participants to rate each task on a scale from 1 to 5, where 5 indicates that LLM assistance is crucial for improving work efficiency, and 1 signifies no impact on task efficiency. In addition, we invited the doctors to propose new tasks across four phases of their workflow, beyond the predefined tasks. Following this, we inquired about the challenges they encounter when using medical LLMs in practice, providing valuable feedback for the development of future medical assistant models. We initially received 82 completed questionnaires. To ensure the validity of the responses, we applied two criteria: (1) the completion time must be more than one-third of the average duration (191.82 seconds) observed across all submissions, indicating potential lack of thoughtful consideration, and (2) responses should not exhibit marked uniformity (e.g., repetitive selection of the same answer option), suggesting insufficient engagement with the content. After applying these criteria, we identified 71 valid responses for analysis. The results revealed that most of the 22 predefined tasks received high ratings, with scores exceeding 4, indicating that LLM assistance is highly effective for these tasks. As shown in Fig. 3, tasks such as *Triage*, *Case Summary*, *Medication Inquiry*, and *Preoperative Education* were rated particularly highly. Doctors found medical LLM assistance in these tasks especially valuable due to their repetitive nature (e.g., *Case Summary, Preoperative Education*), relatively low medical risk (e.g., *Triage*), and high information demands (e.g., *Medication inquiry*). None of the tasks was proposed by more than five respondents, reinforcing that the final set of 22 tasks is widely applicable and relevant across the surveyed doctors. Among the participants, 46.5% reported using LLMs to assist with their clinical work. When asked about the limitations of current medical LLM capabilities, respondents showed strong consensus on several issues. Specifically, 42.2% of doctors identified problems with noncompliance to instructions, 48.5% reported instances of incorrect answers, and 39.4% expressed concerns about the LLM’s inability to provide accurate references. Additionally, doctors emphasized the necessity of continuously updating the LLM’s knowledge base and incorporating self-correction mechanisms to improve the reliability and accuracy of the model’s outputs.

### Task comparison between typical medical datasets and our defined tasks

**Fig. 4:**
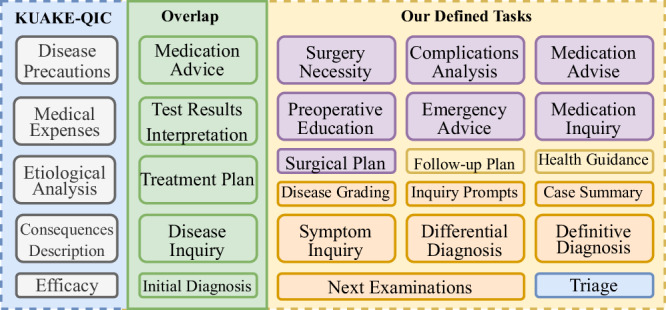
Task overlap between our defined tasks and KUAKE-QIC, highlighting the 17 unique tasks introduced in our framework.

We further compared the tasks defined in our framework with those in typical medical datasets, using KUAKE-QIC^18^ as a representative example. While some overlap exists between the datasets, our defined tasks introduce 17 additional tasks not covered by KUAKE-QIC, highlighting the broader scope and versatility of our approach, as illustrated in Fig. 4.

### DoctorFLAN construction

**Fig. 5:**
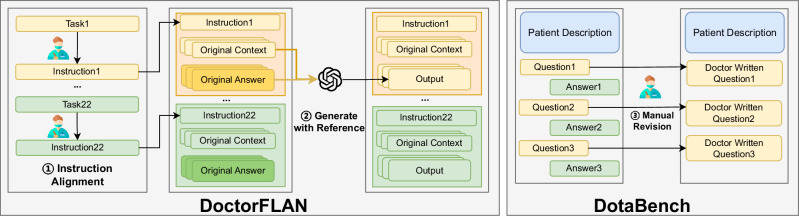
Reference-enhanced refinement in DoctorFLAN and DotaBench.

To create a comprehensive dataset covering the entire clinical workflow, we constructed single-turn DoctorFLAN based on the 22 predefined tasks. First, we collected raw medical data from a variety of sources, then we heuristically filtered and map the data to the relevant tasks. The dataset was refined in two stages: instruction normalization and response polishing. Following the initial construction, we conducted manual verification of a subset of the data by medical experts to ensure its quality, as shown in Fig. 5.

#### Data source

We used three primary data sources: medical multiple-choice questions (MCQs) (e.g., https://www.medtiku.com/), medical encyclopedia entries (e.g., https://m.120ask.com/), and high-quality existing medical datasets such as PromptCBLUE^29^. MCQs are chosen for their ability to simulate a broad range of clinical scenarios, making them highly relevant to real-world practice. The medical encyclopedia, which contains detailed information on topics such as drugs and symptoms, provides a comprehensive and reliable reference, especially for tasks like *Medication Inquiry*. Additionally, we include overlapping datasets from resources like the *Case Summary* subset in PromptCBLUE.

#### Preprocessing and task mapping

After collecting the raw data, we performed deduplication using Jaccard similarity (threshold = 0.8) to eliminate near-duplicate entries and improve data quality^36^. We then categorized the data into the 22 predefined task types using a carefully designed set of task-specific regular expressions. Each task was associated with multiple regex patterns, which are iteratively refined based on expert feedback. In each iteration, we sampled 50 examples for manual annotation by a senior physician to assess classification quality. The refinement process continued until the regex-based categorization achieves over 95% agreement with expert labels, ensuring high precision and consistency. The description of the regex process and an example for task classification are provided in Section A of the Supplementary Information.

#### Reference-enhanced refinement

Although we have gathered data for the 22 tasks, the initial dataset contained issues such as poorly worded instructions and overly brief responses. To address these problems, we implemented a two-step refinement process: instruction alignment and response polishing. In the instruction alignment phase, medical professionals were enlisted to manually draft task-specific instructions for each of the 22 tasks, ensuring that the instructions accurately reflect real-world clinical scenarios and align with the intended task. In the response polishing phase, we asked GPT-4 to generate more comprehensive responses by referencing the original data to enhance their quality. The final dataset contains 92350 samples, divided into a training set DoctorFLAN-*train* and a test set DoctorFLAN-*test*. The test set includes 25 randomly sampled entries from each task, for a total of 550 samples.

To ensure that the responses generated by GPT-4 are factually accurate and realistic, we used a structured review process in which a sample of 1050 responses (50 samples per item across 22 tasks) was reviewed by three medical professionals, each reviewing 350 items. The verification process was overseen by a senior expert with a high-level title, who dedicated 10 hours to ensure a thorough assessment. Each model response was reviewed alongside its corresponding reference answer, and the reviewers are instructed to revise or refine the outputs as needed based on that reference. Rather than conducting blind, independent annotation, this process was designed as a reference-grounded refinement task aimed at improving factual correctness and clinical appropriateness. This approach balances thoroughness with practical limitations, ensuring credible verification within the available resources. The verification criteria include *Correctness*, where a response is considered correct if it contains no factual errors, and *Practicality*, where a response is deemed practical if it is more effective than the original answer. Our results demonstrate correctness (100%) and practicality (99.9%), underscoring the robustness of the DoctorFLAN. In a detailed examination of the data verification stage, we identified an instance where a doctor noted the lack of practicality, commenting on the “lack of specific details," as shown in Table 8. Such feedback suggests that the responses refined by GPT-4 can sometimes fall short in complex practical medical contexts, highlighting an area for future improvement.

**Table 8:** The content presented has been translated from Chinese: an example of a response judged by doctors to be less practical compared to the original, illustrating a potential challenge in ensuring the utility of LLM-generated content in medical data

Instruction:
Facial pain: Provide a detailed overview of the symptoms, possible causes, and related diseases. Include the definition of the symptoms, typical manifestations, common causes that may cause the symptoms, and other health issues related to these symptoms.
Reference:
associated_diseases: Infection in the infratemporal space, chronic maxillary sinusitis, temporal arteritis, infection under the lower jaw, infection in the submandibular space, osteomyelitis of the jaw, bone cyst, petrositis of the temporal bone, acute osteomyelitis of the jaw, acute submandibular sialadenitis
overview: Facial pain refers to pain on one or both sides of the face, or at the forehead.
This pain can be dull or throbbing and can be extremely sharp.Facial pain or spasms, generally not life-threatening, but the sufferer is very miserable; even if the pain is bearable, the facial expression appears unnatural.
cause: Facial pain is usually caused by local tissue infection or inflammation; some are throbbing pain, some are severe stabbing pain, and the presented symptoms vary. Muscle inflammation is the most familiar cause of facial pain, also known as temporomandibular joint disorder. Followed by trigeminal neuralgia, herpes zoster. It can also be seen in facial pain caused by oral inflammation, and pain caused by local trauma.
Answer:
Facial pain is a relatively common clinical symptom, characterized by various forms and manifestations. Patients may describe facial pain as dull pain, throbbing pain, burning sensation, or extreme stabbing pain. When considering the causes of facial pain, it is necessary to comprehensively consider the distribution, nature, triggering and alleviating factors, and accompanying symptoms.
According to < textbook knowledge >, diseases associated with facial pain include, but are not limited to, infection in the infratemporal space, chronic maxillary sinusitis, temporal arteritis, infection under the lower jaw, infection in the submandibular space, and osteomyelitis of the jaw.
Doctor comment:
The answer lacks specific detail compared to the original reference answer.

### DotaBench construction

Extending the single-turn dataset DoctorFLAN, we introduced multi-turn DotaBench to evaluate multi-turn dialogues involving medical assistants. This extension is motivated by the need to assess an LLM’s ability to operate in realistic clinical settings, where conversations often span multiple turns and involve a sequence of logically connected questions. While DoctorFLAN captures isolated queries, DotaBench focuses on multi-turn interactions in which each question is designed to build upon the previous one, simulating the stepwise inquiry process commonly used by physicians in real-world consultations.

#### Data source

To ensure clinical authenticity, we selected CMB-Clin^10^ as the source corpus. CMB-Clin is a multi-round question-answering dataset derived from real medical records. However, its original format consists of 2–4 standalone Q&A pairs that lack contextual continuity, making it unsuitable for dialogue-based evaluation in its raw form.

#### Reference-enhanced refinement

To address this limitation, we worked with licensed physicians to manually restructure the data into coherent three-turn dialogues. Specifically, we extracted key clinical elements from each case, such as chief complaints, physical findings, and diagnostic test results, and asked physicians to reformulate them into contextually connected questions that reflect realistic consultation workflows. The original answers from CMB-Clin are retained as reference responses, which are later used to support reference-based evaluation under the LLM-as-a-judge framework. A representative example illustrating this transformation is included in Supplementary Tables 1 and 2. Unlike DoctorFLAN, which directly involves LLMs in data generation, DotaBench is crafted without LLM intervention, thereby eliminating the need for subsequent data verification and ensuring controlled evaluation conditions.

### Data statistic

The statistical analysis of the DoctorFLAN and DotaBench datasets is presented in Table 9. The DoctorFLAN dataset comprises 92,326 instances across 22 distinct tasks, involving 27 medical specialties in total as detailed in Table 10, demonstrating the comprehensive coverage of DoctorFLAN in real clinical scenarios. In addition, we extracted a subset of 25 instances from each task, referred to as DoctorFLAN-*test* for evaluation. The training and test sets are created via a random split. The DotaBench dataset includes 74 instances of 3-turn conversations.

**Table 9:** The statistics of DoctorFLAN and DotaBench dataset (the asterisk indicates that the number of samples per task varies)

	DoctorFLAN		DotaBench
Type	Single-turn		3-turns
Split	train	test	test
Specialist	27	27	-
Task	22	22	-
#Q/task	-*	25	-
#Q in total	91,776	550	74

**Table 10:** Specialists and tasks in the DoctorFLAN dataset

Specialist	Gastroenterology, Pediatrics, Obstetrics & Gynecology, Respiratory,
	Medicine, Cardiology, Neurology, General Surgery, Stomatology, Nephrology,
	Hepatology, Orthopedics, Urology, Spine Surgery, Cardiothoracic Surgery,
	OphthalmologyHematology, Endocrinology, Oncology, Emergency Medicine,
	Infectious Disease, Traditional Chinese MedicineRheumatology & Immunology,
	Neurosurgery, Dermatology, Otorhinolaryngology (ENT), Vascular Surgery,
	Multidisciplinary
Task	Pre-Diagnosis: Triage
	Diagnosis: Inquiry Prompts, Symptom Inquiry, Disease Inquiry, Initial Diagnosis,
	Case Summary, Differential Diagnosis, Next Examinations, Test Results Interpretation,
	Definitive Diagnosis, Disease Grading
	Treatment: Emergency Advice, Treatment Plan, Medication Inquiry,
	Medication Advice, Complications Analysis, Treatment Adjustment,
	Surgery Necessity, Surgical Plan, Preoperative Education
	Post-Treatment: Health Guidance, Follow-up Plan

### Model training

We fine-tuned two open-source backbone models, Yi-6B and Baichuan2-7B-Base, using a standard supervised fine-tuning (SFT) framework with an autoregressive, decoder-only architecture. To ensure the model captures both domain-specific expertise and general ability, we constructed a mixed training corpus comprising 92k task-aligned medical samples from DoctorFLAN, 101k general-purpose instruction samples from datasets such as Evol-instruct^37^, ShareGPT^38^, and 51k additional medical QA pairs from CMExam^27^.

All models were trained on 4 NVIDIA A100 GPUs. We set the maximum input sequence length to 4096 tokens and used a per-GPU batch size of 4, training for 3 epochs with a learning rate of 5 × 10^−5^. The optimization used the AdamW optimizer with decoupled weight decay, and gradient checkpointing was enabled to reduce memory consumption. Mixed precision training was performed using fp16 format to accelerate computation.

The objective function is the negative log-likelihood (NLL) of the target response given the prompt, encouraging the model to generate accurate and fluent outputs aligned with medical task instructions. Specifically, the loss is defined as:

1 $${{\mathcal{L}}}_{{\rm{SFT}}}=-\mathop{\sum }\limits_{t=1}^{T}\log P({y}_{t}| x,{y}_{ < t})$$

where *x* denotes the input prompt and *y*_*t*_ the target token at time step *t*. The final model checkpoint was selected after three training epochs based on manual review and preliminary validation performance, without using early stopping or automated selection heuristics.

### Evaluation models

To comprehensively evaluate the performance of medical-specific models trained on various backbones and datasets, we assessed a wide range of Chinese medical LLMs on DoctorFLAN-*test* and DotaBench.

Among the domain-specific models, we included BianQue-2^8^, a medical model fine-tuned from ChatGLM-6B^39^ using patient-doctor dialogs; DISC-MedLLM^33^, a model based on the Baichuan-13B-Base architecture designed for deep medical interactions; HuatuoGPT-7B^6^, fine-tuned from Baichuan-7B for Chinese medical consultation; and HuatuoGPT-II-7B^5^, a state-of-the-art medical LLM built on Baichuan2-7B with extensive medical knowledge.

We also evaluated general-purpose models to provide a performance baseline. These included Qwen-1.8B-Chat^40^, fine-tuned with SFT and reinforcement learning with human feedback; Baichuan-13B-Chat^41^, which shares the same backbone as DISC-MedLLM and demonstrates strong general performance; and Baichuan2 models, including Baichuan2-7B-Chat and Baichuan2-13B-Chat^42^. We further include Yi-6B-Chat and Yi-34B-Chat^43^, which represent two scales of models from the Yi series, comparable to Qwen and Baichuan.

To broaden the comparison, we additionally report results from proprietary models such as GPT-3.5, GPT-4, and Claude-3.

All models are evaluated using the same decoding hyperparameters: max_new_tokens = 1024, top_p = 0.7, temperature = 0.5, and repetition_penalty = 1.1. We adopt CoT prompting, without using any additional augmentation techniques.

### Evaluation method

Considering both accuracy, reliability, and cost, our evaluation methodology incorporates both automatic and human evaluations.

#### Automatic evaluation

Our task involves open-ended answer generation in medical contexts, where multiple correct and clinically valid responses may exist. In such settings, traditional metrics such as BLEU and ROUGE, which rely on N-gram overlap with reference answers, are often inadequate. These metrics fail to capture semantic consistency when answers are phrased differently yet medically equivalent, and are also highly sensitive to variations in response length. To address these limitations, we employed GPT-4 (gpt-4-0125-preview) for automatic evaluation, a method shown to be highly effective in previous research^44^. To ensure evaluation accuracy, we adopted a reference-based model evaluation approach, where the LLM refers to the provided reference and scores responses based on predefined criteria. These scoring standards include: Accuracy (assessing the correctness and reliability of the information), Coherence (evaluating the clarity and logical flow of the responses), Relevance (measuring how closely each response addresses the prompt), and Thoroughness (judging the depth and completeness of the response in covering the topic). During evaluation, we applied Chain-of-Thought (CoT) prompting both in response generation and in the LLM-as-a-judge scoring process. We did not use any external augmentation techniques, such as retrieved rationales or tool-assisted reasoning. The evaluation was performed using GPT-4, accessed via the official OpenAI API with default inference settings. To support reproducibility, we provided the full evaluation prompt in Supplementary Figs. 1 and 2.

To balance accuracy and resource constraints, we conducted human evaluation on a subset of models. For DoctorFLAN-*test*, which contains 550 questions in total, we divided them into six roughly equal parts, with 91 or 92 questions per evaluator. Each evaluator was assigned a set of questions and tasked with rating the responses of all six models for each question, ensuring a fair and consistent evaluation across all models. The evaluation team consists of six healthcare professionals with varying levels of experience: three mid-level professionals with 5–6 years of experience, two associate senior professionals with 12 years of experience, and one senior professional with 26 years of experience. Evaluators are compensated based on their professional seniority, with senior professionals receiving an hourly rate of 250 RMB, while mid-level professionals were paid 165 RMB per hour. For DotaBench, we invited three doctors to participate in the evaluation process, with each spending an average of 3 hours reviewing the data.

## Supplementary information

Supplementary information

## Data Availability

DoctorFLAN and DotaBench datasets used in this study are available at https://huggingface.co/datasets/FreedomIntelligence/DoctorFLAN and https://huggingface.co/datasets/FreedomIntelligence/DotaBench, respectively. The source code for DotaGPT training and evaluation is available at https://github.com/FreedomIntelligence/DotaGPT.

## Code availability

The source code for DotaGPT training and evaluation is available at https://github.com/FreedomIntelligence/DotaGPT.
