# Supplementary material for: Enabling doctor-centric medical AI with LLMs through workflow-aligned tasks and benchmarks
Source: Npj Health Syst. 2025 Nov 25;2:44. doi: 10.1038/s44401-025-00038-z (PMC13354162; doi:10.1038/s44401-025-00038-z)
Supplement: Supplementary file 1 — Supplementary information [file 44401_2025_38_MOESM1_ESM.pdf]

A Regex-Based Data Categorization

Note: Since our QA corpus is in Chinese, the regex patterns are expressed using Chinese characters where necessary. Each pattern is accompanied by an English explanation for clarity.

To enable efficient and scalable preprocessing, we employ regular expression (regex) rules to automatically filter and categorize medical QA samples. The overall pipeline consists of two stages: (1) general data cleaning and (2) task-specific classification using field-wise regex matching.

General Filtering.

Before task categorization, we applied two regex-based filters to the entire dataset:

- **Case-based QA Selection:** We retained only questions that involve real-world case descriptions, identified by matching sentence openings such as "患者", "男", "女", "患儿", or "某患者".
- **Exclusion of Image/Table-Based Questions:** We removed samples referencing visual content using the following regex pattern:

Regex Rule for Filtering Text-Based Content

```
r'如图|结合图像|图像如下|img|图示|图[0-9]+|表[0-9]+'
```

Example: Category Classification via Multi-Field Regex Matching.

Each QA item was decomposed into five semantic fields to support modular rule design:

1. Case description
2. Question content
3. Metadata (e.g., subject area such as "传染病学" / infectious diseases)
4. Answer text
5. Answer Option Set

To classify data into specific clinical categories, we applied regex rules over **multiple fields in combination**, instead of relying on any single field alone.

For instance, to extract QA samples related to *Differential Diagnosis* task, we used the following logic:

- **Field 2 (Question Content):** We matched the question text against a regex that detects diagnostic comparison terms (e.g., "鉴别", "诊断", "区别"), while filtering out questions focused on treatment, symptoms, or procedures. The regex used was:

Regex on Question Content

```
r'^(?!.*(?:明确|检查|并发症|意见|利于|必备条件|鉴别要点|治疗|问题|项目|体征|条件))(?=.*(?:鉴别|诊断|区别)).*$'
```

Prompt	Question
Turn 1	这位49岁的男性病人在3小时前解大便后出现右下腹疼痛，他自己可以触及右下腹的一个包块。他之前都很健康，没有什么特别的既往史。你能帮我根据这些信息给出一个初步的诊断吗? (Translated) This 49-year-old male patient developed right lower abdominal pain three hours ago after a bowel movement, and he can palpate a lump in the same area. He has no notable past medical history.Based on this information, could you provide a preliminary diagnosis?
Turn 2	我刚刚进行了体格检查，发现他体温37.8℃，心率101次／分，呼吸22次/分，血压100/60mmHg。于右侧腹股沟区可扪及一圆形肿块约4cm×4cm大小，并有压痛、界欠清，在腹股沟韧带上内方。请问还需要哪些辅助检查来确定诊断? (Translated) I just performed a physical examination. The patient has a temperature of 37.8°C, a heart rate of 101 bpm, respiratory rate of 22 breaths/min, and blood pressure of 100/60 mmHg. A round mass approximately 4 cm × 4 cm is palpable in the right inguinal region, with tenderness and poorly defined borders. It is located medial to the inguinal ligament. What additional diagnostic tests would you recommend to confirm the diagnosis?
Turn 3	检验结果出来了。血常规显示白细胞计数5.0×109／L，中性粒细胞78%。尿常规正常。多普勒超声检查沿腹股沟纵切可见一多层分布的混合回声区，宽窄不等，远端膨大，边界整齐，长约4～5cm。腹部X线检查可见阶梯状液气平。根据这些信息，请帮我明确诊断，并提供治疗方案。 (Translated) The test results are now available. The complete blood count shows a white blood cell count of 5.0×10/L with 78% neutrophils. Urinalysis is normal. Doppler ultrasound reveals a multilayered mixed-echo region along the longitudinal section of the inguinal area, with uneven width and distal enlargement, measuring approximately 4–5 cm with well-defined borders. Abdominal X-ray shows a step-ladder pattern of air-fluid levels. Based on this information, could you confirm the diagnosis and recommend a treatment plan?

Supplementary Table 1: A manually constructed DotaBench example consisting of three contextually linked turns that reflect realistic consultation workflows.

- **Field 5 (Answer Option Set):** We required that the question includes more than one answer option, as differential diagnosis questions often present multiple candidate conditions for selection.

Only when both conditions were satisfied, namely that relevant keywords appeared in the question and that multiple answer options were present, did we label the sample as belonging to the *Differential Diagnosis* category.

This combination-based rule design ensures higher precision and flexibility, and can be extended to other categories by customizing field-specific regex patterns.

B Dotabench Construction Details

Supplementary Tables 1 and 2 present an example of how we convert a raw case from CMB-Clin into a contextually linked multi-turn consultant sample in DotaBench.

C Evaluation Prompt

Supplementary Figures 1 and 2 show the complete prompt templates used for DoctorFLAN and DotaBench, respectively.

Case Description	现病史（1）病史摘要 病人，男，49岁，3小时前解大便后出现右下腹疼痛，右下腹可触及一包块，既往体健。（2）主诉 右下腹痛并自扪及包块3小时。 体格检查 体温： T 37.8℃，P 101次／分，呼吸22次/分，BP 100/60mmHg，腹软，未见胃肠型蠕动波，肝脾肋下未及，于右侧腹股沟区可扪及一圆形肿块，约4cm×4cm大小，有压痛、界欠清，且肿块位于腹股沟韧带上内方。辅助检查（1）实验室检查 血常规：WBC 5.0×109／L，N 78%。 尿常规正常。 （2）多普勒超声检查 沿腹股沟纵切可见一多层分布的混合回声区，宽窄不等，远端膨大，边界整齐，长约4～5cm。（3）腹部X线检查可见阶梯状液气平。<br>(Translated) Present Illness History: (1) Summary: A 49-year-old male developed right lower abdominal pain three hours ago after defecation, with a palpable mass in the same area. No significant medical history. (2) Chief Complaint: Right lower abdominal pain and a self-palpated mass for 3 hours. Physical Examination: Temperature: 37.8°C, Pulse: 101 bpm, Respiration: 22/min, Blood Pressure: 100/60 mmHg. Abdomen soft, no visible peristaltic waves, liver and spleen not palpable. A round mass (4×4 cm) with tenderness and poorly defined borders is palpable in the right inguinal region, medial to the inguinal ligament. Auxiliary Tests: (1) Laboratory Tests: CBC: WBC 5.0×10/L, Neutrophils 78% Urinalysis: Normal (2) Doppler Ultrasound: Multilayered mixed-echo area along the inguinal longitudinal section with variable width and distal enlargement; well-defined borders; 4–5 cm in length (3) Abdominal X-ray: Step-ladder air-fluid levels observed
Question 1	简述该病人的诊断及诊断依据。<br>(Translated) Summarize the diagnosis and diagnostic rationale.
Question 2	简述该病人的鉴别诊断。<br>(Translated) Summarize the differential diagnosis.
Question 3	简述该病人的治疗原则。<br>(Translated) Summarize the treatment principles.

Supplementary Table 2: The original CMB-Clin case record used as the source for DotaBench construction. Note that the original QA pairs are isolated and lack multi-turn context.

Evaluation Prompt for DoctorFLAN-*test*

System Prompt:

Please act as an impartial judge and evaluate the quality of the response provided by an AI assistant to the user question displayed below.
Requirements: Your assessment should focus primarily on the consistency between the assistant's answer and the reference answer.
Begin your evaluation by providing a short explanation. Be as objective as possible. After providing your explanation, you must rate the response on a scale of 1 to 10 by strictly following this format: "[[Rating]]", for example: "Rating: [[5]]".

Prompt:

[Question]
{question}

[The Start of Reference Answer]
{reference}
[The End of Reference Answer]
[The Start of Assistant's Answer]
{answer}
[The End of Assistant's Answer]

Supplementary Figure 1: Evaluation Prompt for DoctorFLAN-*test*.

Evaluation Prompt for DotaBench

System Prompt:

Please act as an impartial judge and evaluate the quality of the response provided by an AI assistant to the user question displayed below.
Requirements: Your assessment should focus on the overall quality of the responses based on the following criteria:
Accuracy: Evaluate the correctness and reliability of the information provided. Coherence: Assess the clarity and logical flow of the responses. Relevance: Determine how closely each response addresses the question asked. Thoroughness: Judge the depth and completeness of the response in covering the topic.
You will be given the assistant's answer and some references. The reference consists of Q&A pairs related to the patient, which are completely accurate and can be used as a reliable source of truth.
You evaluation should focus on the assistant's answer to the first question. Begin your evaluation by providing a short explanation. Be as objective as possible. After providing your explanation, you must rate the response on a scale of 1 to 10 by strictly following this format: "[[Rating]]", for example: "Rating: [[5]]".

Prompt:

<|The Start of Reference|>

{reference}

<|The End of Reference|>

<|The Start of Assistant A's Conversation with User|>

User:
{question_1}

Assistant A:
{answer_1}

<|The End of Assistant A's Conversation with User|>

Supplementary Figure 2: Evaluation Prompt for DotaBench
